# Expression of *DLK1* and *MEG3* genes in porcine tissues during postnatal development

**DOI:** 10.1590/s1415-47572010000400030

**Published:** 2010-12-01

**Authors:** Maria Oczkowicz, Agata Piestrzyska-Kajtoch, Katarzyna Piórkowska, Barbara Rejduch, Marian Rózycki

**Affiliations:** 1 Department of Genetics and Animal Breeding, National Research Institute of Animal Production, BalicePoland; 2 Department of Immuno and Cytogenetics, National Research Institute of Animal Production, BalicePoland

**Keywords:** *DLK1*, imprinting, *MEG3*, pigs, polar overdominance

## Abstract

The *Drosophila*-like homolog 1 (DLK1*)*, a transmembrane signal protein similar to other members of the Notch/Delta/Serrate family, regulates the differentiation process in many types of mammalian cells. Callipyge sheep and *DLK1* knockout mice are excellent examples of a fundamental role of the gene encoding DLK1 in muscle growth and fat deposition. *DLK1* is located within co-regulated imprinted clusters (the *DLK1/DIO3* domain), along with other imprinted genes. Some of these, *e.g.* the RNA coding *MEG3* gene, presumedly interfere with *DLK1* transcription. The aim of our study was to analyze *DLK1* and *MEG3* gene expression in porcine tissues (muscle, liver, kidney, heart, brain stem) during postnatal development. The highest expression of both *DLK1* and *MEG3* variant 1 *(MEG3 var.1)* was observed in the brain-stem and muscles, whereas that of *MEG3* variant 2 *(MEG3var.2)* was the most abundant in muscles and the heart. During development (between 60 and 210 days of age) expression of analyzed genes was down-regulated in all the tissues. An exception was the brain- stem, where there was no significant change in *MEG3* (both variants) mRNA level, and relatively little decline (2-fold) in that of *DLK1* transcription. This may indicate a distinct function of the *DLK1* gene in the brain-stem, when compared with other tissues.

The *Drosophila*-like homolog 1 (DLK1) is a transmembrane signal protein similar to other members of the Notch/Delta/Serrate family (Jensen *et al.*, 1994). This protein controls several cell-differentiation processes throughout embryonic and adult life. A role of DLK1 in adipogenesis has been well documented (Smas and Sul, 1996; Sul , 2009; Nueda *et al.*, 2007). Recently, DLK1 was shown to regulate fate of myogenic cells (Andersen *et al.*, 2009) and human skeletal stem cells (Abdallah *et al.*, 2004). A significant role of *DLK1* in maintaining proper organism function was demonstrated by generating *DLK1* knockout-mice, which exhibited accelerated obesity, growth disorders and skeletal malformation (Moon *et al.*, 2002). Nevertheless, knowledge about exact function of *DLK1* in particular tissues and organs remains fragmentary.

A gene for *DLK1* is located in imprinted gene clusters on chromosomes 12, 14 and 7 within the so-called *DLK1/DIO3* domain of mice, humans and pigs, respectively. There are few imprinted genes in the neighborhood of *DLK1*, among which the best studied are paternally expressed *PEG11/RTL1*, maternally expressed *MEG3/GTL2* and *MEG8.* The full-length PEG11 protein was recently identified in callipyge sheep muscle (Byrne *et al.*, 2010). On the other hand, *MEG3* and *MEG8* genes produce non-coding RNAs (ncRNA). These play an essential role in growth and differentiation, as the deletion of *MEG3/GTL2*, together with its differentially methylated region, induces lethal parent-origin-dependent defects in mice (Takahashi *et al.*, 2009). The whole domain has been intensively studied in reference to molecular mechanism of imprinting control, but to date no satisfactory model has been proposed for this cluster.

Among farm animals, the most extensive studies of the *DLK1/PEG11* domain have been undertaken in sheep, mainly in an attempt to identify the Single Nucleotide Polymorphism (SNP^CLPG^) responsible for the so called “callipyge phenotype” in this region. The callipyge phenotype (muscle hypertrophy of the hindquarters) is inherited in a non-Mendelian manner (the so-called polar overdominance), and appears in the offspring only when the mutated allele comes from the father and the wild one from the mother (C^pat^/N^mat^ genotype) (Cockett *et al.*, 1996). Animals with other genotypes (C^pat^/C^mat^ N^pat^/N^mat^, N^pat^/C^mat^) do not exhibit the characteristic phenotype. There is a theory explaining the molecular mechanism underlying the unusual inheritance of callipyge phenotype: *CLPG* mutation enhances the level of *DLK1*, *MEG3*, *PEG11* and *MEG8* gene expression in *cis*, probably by modifying the activity of a common regulatory element. The callipyge phenotype results from a *DLK1* and/or *PEG11* overexpression in skeletal muscle with simultaneous underexpression of *MEG3* and *MEG8.* In C^mat^/C^pat^ individuals, overexpression of *MEG8* and *MEG3* or other maternally expressed genes interfere in trans with *DLK1* and *PEG11* and inhibit expression of *DLK1* and *PEG11* (Charlier *et al.*, 2001; Georges *et al.*, 2003).

The considerable effect of SNP^CLPG^ on sheep musculature has made *DLK1* an interesting candidate gene for marker-assisted selection in other farm animals. Unfortunately, SNP^CLPG^ appeared to be a private allele, encountered exclusively in callipyge flocks (Smit *et al.*, 2003). Nevertheless, Kim *et al.* (2004), on identifying a *DLK1* polymorphism (silent SNP mutation) associated with growth, fatness and body composition in pigs, proved its polar-overdominant inheritance. These results have been confirmed through QTL (Quantitative Trait Locus) analysis, although the molecular mechanism involved has, as yet, not been stated (Li *et al.*, 2008). Recently, *gDLK1* has been proposed as a novel selection-marker for high muscle growth in chickens, since observed *DLK1* mRNA expression was greater in the muscles of broilers than in layers (Shin *et al.*, 2009).

In pigs, four different *DLK1* and two *MEG3* transcripts have been identified, so far (Deiuliis *et al.*, 2006; Li *et al.*, 2008; Samulin *et al.*, 2009). In the pigs, the short form (*DLK1* C2) is the most abundant transcript in all analyzed tissues (Deiuliis *et al.*, 2006, Samulin *et al.*, 2009). *MEG3* expression has recently been observed in the liver, heart, spleen, fat, kidney and skeletal muscle of two-month old pigs. Nevertheless, relative abundance of different *MEG3* variants has not been studied so far (Jiang and Yang, 2009).

Since *DLK1* appears to be an interesting candidate gene for marker-assisted selection, and as little is known about postnatal gene expression within the *DLK1/DIO3* domain in pigs, we decided to analyze relative mRNA abundance of *DLK1* and *MEG3* genes in various porcine tissues (muscle, liver, brain, kidney, heart), as well as changes in the expression of these genes during postnatal development.

Animals for the study were kept in the Pilot Plant of the National Research Institute of Animal Production in Pawlowice under identical housing and feeding conditions. They were divided into 6 age-groups, according to the day of slaughter (60-, 90-, 120-, 150-, 180- and 210-days-old pigs). In the first part of the experiment, two batches each of the breeds Large White and Duroc, with 4 to 6 pigs of both in each age group, were analyzed separately. As no significant differences between breeds were found, we decided to create only age groups consisting of 8 to12 animals (4 to 6 Large White and 4-6 Duroc). Animals were related – all pigs within the breed had the same father, and their mothers were sisters. Fragments of muscle *(longissimus dorsi)* and the liver were collected from animals of all the age groups, whereas fragments of the brain-stem, heart and kidney were collected only from the 60 and 210 days groups. Tissues were collected immediately after slaughter, and kept in liquid nitrogen during transportation. All animals were stress-resistant (RYR1 C/C).

Total RNA was extracted using TRI-Reagent (Sigma) and a Silent Crusher S homogenizer (Heidolph), according to the method described by Chomczyski (1993). RNA from the brain-stem was isolated by using the SV Total RNA System (Promega). The amount of extracted RNA was estimated by BioPhotometer (Eppendorf), and its quality evaluated by gel electrophoresis.

The RNA (1 μg) was reverse transcribed into cDNA at 37 °C by using a High Capacity cDNA Reverse Transcription Kit with random primers (Applied Biosystems), according to the manufacturer's protocol.

Primers and probes for all the genes were designed with Primer Express software (Applied Biosystems). *MEG3* primers were designed to distinguish alternative isoforms, whereas *DLK1* primers covered exon-exon junction and amplified all transcripts. The sequences of probes and primers were as follows: *DLK1* for-5'AGGACGGCT GGGATGGA, rev-5'CGAGGTTCGCGCAGGTT, probe-5'TCTGTGACCTAGACATC *MEG3 var. 1* for-5'GG AAGGGACCTCAGACATGTG, rev-5'CCTAGCCTGC CGATTTCAGA, probe-5'CTCCTGCACCCTCC, *MEG3 var. 2* for- 5'TGAGGCTGGAGGAGCGTTAG, rev-5'CCAACTTGGACCCCTTCTCTT, probe-5'ATCTTGT CGCATTGCT.

Relative quantification of expression was performed on a 7500 Real-Time PCR System using labeled TaqMan® Tamra and MGB probes and TaqMan® Universal PCR Master Mix with UNG AmpErase (Applied Biosystems). Reactions, in a total volume of 25 μL were done in triplicate and according to the TaqMan® Universal PCR Master Mix protocol*. GAPDH* was used as endogenous control to compare expression changes of *DLK1* and *MEG3* during development in muscle and liver. *RPL27* was used for between-tissue comparison, due to variations in *GAPDH* expression among the analyzed tissues. The results were analyzed using Sequence Detection System software v. 2.0 (Applied Biosystems). Statistical analysis was performed using One Way Anova, Tukey test (SAS Institute).

In our investigation we compared relative expression of *DLK1* and *MEG3* genes in the brain stem, muscle, heart, kidney and liver of pigs. For the muscle (*longissimus dorsi*) and liver, six developmental stages (60, 90, 120, 150, 180 and 210 days) were taken into consideration, whereas for the brain-stem, heart and kidney only two were (60 and 210 days).

The lowest expression of *DLK1* and *MEG3 var. 1* was noted in the liver, and the highest in the brain-stem (*DLK1* – 3.8-fold higher than in liver, *MEG3 var.1* - 26-fold higher). An exception was the *MEG3 var.2* transcript, which was the most abundant in the muscle (23-fold higher than in liver). In the muscle, abundance of other transcripts was also relatively high (*DLK1* - 3-fold higher than in liver, *MEG3 var. 1* – 14-fold higher). The level of expression was intermediate in the heart and kidneys, when compared with other tissues, although *MEG3* transcripts were more abundant in the heart than in the kidney, whereas in *DLK1* transcripts this was to the contrary (Figure 1).

We also analyzed alteration in the expression of these genes during postnatal development. In the brain stem, kidney and heart *DLK1* and *MEG3* mRNA abundance was compared between 60 and 210 days of age, while in the muscle and liver additional developmental periods were analyzed (90, 120, 150 and 180 days of age). The expression level of all transcripts decreased with age in all the tissues investigated (Figures 1  and 2), with the exception of *MEG3* (both variants) in the brain, where there was no significant change. *DLK1* gene expression declined much more intensively (2-fold in the brain between 60 and 210 days, 8-fold in the heart, 14-fold in the liver and 12-fold in the muscle) than *MEG3* (both transcripts, 1.4-, 2.6-, 2,2-fold higher in the respective tissues). In the brain-stem, expression of the analyzed genes seemed to be more stable during aging, when compared to the other analyzed tissues (Figure 1). In the muscle, where more developmental stages were analyzed, no fluctuation during the 90-180-days interval was observed. *DLK1* expression decreased gradually, whereas that of *MEG3* remained similar at 60 and 90 days to afterwards enter in sharp decline (Figure 2a). The pattern of DLK1 expression in the liver was similar to that observed in the muscle, whereas that of *MEG3* was more pronounced only at 60 days (Figure 2b).

Previous studies have shown that in humans, *DLK1* gene expression is high during prenatal development, whereas after birth this is restricted to hormone-secreting cells and monoaminergic neurons in the central nervous system (Abdallah *et al.*, 2007). In contrast, postnatal *DLK1* expression in pigs was observed in the kidneys, heart, spleen, fat and muscles (Deiuliis *et al.*, 2006), as confirmed herein. Moreover, high mRNA abundance of *DLK1* and *MEG3* in the brain-stem was detected, with the *MEG3 var.1* being the predominant variant of the latter. To date, *DLK1* expression has not been studied in the brain-stem of farm animals, although in adult mice, high *DLK1* and *MEG3* expression has been observed in certain regions of the brain, *e.g.* hypothalamus, medulla and cerebral cortex (www.brain-map.org) (Labialle *et al.*, 2008). A semi-quantitative analysis of tissue-specific expression of porcine *MEG3* gene has been performed previously by RT-PCR (Jiang and Yang, 2009).The authors noted the highest expression of this gene in the brain and lungs, followed by the tongue, spleen, stomach, fat, kidneys, liver, skeletal muscles, heart and small intestine. Contrarily, our results suggest a much higher expression of *MEG3* (both variants) in skeletal muscles than in the liver or kidneys. However, the results are difficult to compare due to the multiple isoforms of the *MEG3* gene.

**Figure 1:**
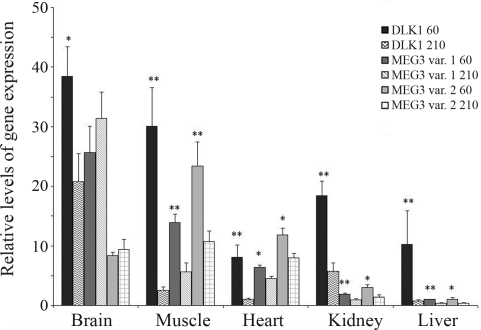
*DLK1*, *MEG3* var.1 and *MEG3* var. 2 gene expession in various porcine tissues at 60 and 210 days after birth. Values are presented as mean ± SEM. Asterisks indicate significant differences between 60 and 210 days for each gene. *p < 0.05, **p < 0.01.

**Figure 2:**
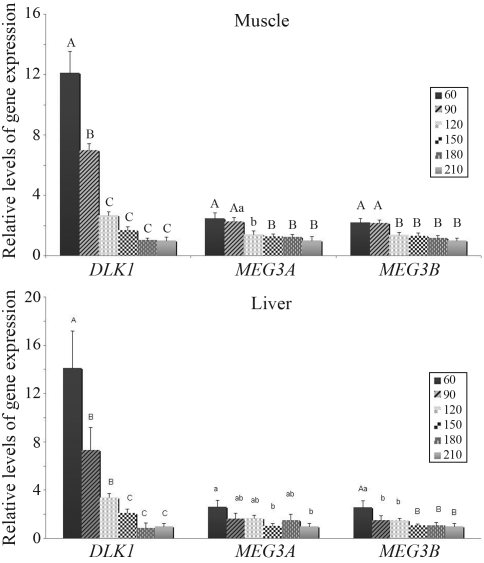
*DLK1*, *MEG3* var.1 and *MEG3* var. 2 gene expression in the *longissimus dorsi* muscle (a) and liver (b) of pigs at 60, 90, 120, 150, 180 and 210 days after birth. Values are presented as mean ± SEM. Different letters indicate significant differences. Capital letters – p < 0.01, small letters – p < 0.05.

Declining expression of eleven imprinted genes (including *DLK1* and *MEG3*) during postnatal development in multiple tissues (heart, lung and kidney) of mice at 1, 4 and 8 weeks of age has been previously reported (Lui *et al.*, 2008). The authors inferred that the down-regulation of imprinted genes contributes to a deceleration in organ growth. Recently *DLK1* has been shown to regulate growth hormone (*GH)* expression (Ansell *et al.*, 2007). This, as well as our results, strongly supports the hypothesis for the kidney, liver and heart. In the present study, the decrease in *DLK1* gene expression was also very pronounced in muscles. This may reflect a deceleration in muscle-mass growth. In contrast, the decline of *DLK1* transcript abundance in the brain was relatively low (2-fold), with no significant change in *MEG3* transcripts, thereby implying the maintenance of high expression levels even at 210 days. This may indicate a distinct function of the *DLK1* gene in the brain-stem.

Although the biological consequences remain unknown, it is evident that expression of the *DLK1/DIO3* domain in pigs, as in mice, is regulated in a tissue-specific manner. A full understanding of the complex mechanism involved in gene expression control in the *DLK1/DIO3* domain, requires an analysis of the imprinting status of genes in various tissues during development. *DLK1* is generally considered as a paternally expressed gene and *MEG3* as a maternally expressed one. An increasing number of experiments, however, imply that the imprinting status of many genes, besides changing during development, differs among tissues (Khatib, 2007). Further studies of expression and the methylation status of this important domain in a wider range of tissues and developmental stages are planned in the future.
